# Human Dermal Stem/Progenitor Cell-Derived Conditioned Medium Ameliorates Ultraviolet A-Induced Damage of Normal Human Dermal Fibroblasts

**DOI:** 10.1371/journal.pone.0067604

**Published:** 2013-07-11

**Authors:** Joong Hyun Shim, Ju-Yearl Park, Mi-Gi Lee, Hak Hee Kang, Tae Ryong Lee, Dong Wook Shin

**Affiliations:** 1 Bioscience Research Institute, Amorepacific Corporation R&D Center, Yongin-city, Gyeonggi-do, Republic of Korea; 2 Hi-Tech Analysis Team, GyeongGi Bio-Center, GyeongGi Institute of Science & Technology Promotion, Suwon-city, Gyeonggi-do, Republic of Korea; Northwestern University Feinberg School of Medicine, United States of America

## Abstract

Adult skin stem cells are considered an attractive cell resource for therapeutic potential in aged skin. We previously reported that multipotent human dermal stem/progenitor cells (hDSPCs) can be enriched from (normal human dermal fibroblasts (NHDFs) using collagen type IV. However, the beneficial effects of hDSPCs on aged skin remain to be elucidated. In the present study, we analyzed the growth factors secreted from hDSPCs in conditioned medium (CM) derived from hDSPCs (hDSPC-CM) and found that hDSPCs secreted higher levels of bFGF, IGFBP-1, IGFBP-2, HGF, VEGF and IGF-1 compared with non-hDSPCs. We then investigated whether hDSPC-CM has an effect on ultraviolet A (UVA)-irradiated NHDFs. Real-time RT-PCR analysis revealed that the treatment of UVA-irradiated NHDFs with hDSPC-CM significantly antagonized the UVA-induced up-regulation of the MMP1 and the UVA-induced down-regulation of the collagen types I, IV and V and TIMP1 mRNA expressions. Furthermore, a scratch wound healing assay showed that hDSPC-CM enhanced the migratory properties of UVA-irradiated NHDFs. hDSPC-CM also significantly reduced the number of the early and late apoptotic cell population in UVA-irradiated NHDFs. Taken together, these data suggest that hDSPC-CM can exert some beneficial effects on aged skin and may be used as a therapeutic agent to improve skin regeneration and wound healing.

## Introduction

Adult stem cells are self-renewable and exist in many adult tissues [1], [2]. These cells are attractive, both because of their potential therapeutic use for replacing damaged cells and because they are crucial to understanding how tissues and organs develop. Mesenchymal stem cells (MSCs), a type of adult stem cells, were initially identified from bone marrow [2]–[4]. MSCs have the potential to differentiate into the mesodermal lineages, such as adipocytes, osteoblasts and chondrocytes, and also non-mesodermal cell types, such as neuronal cells, pancreatic ß cells and hepatic cells [2]–[10]. Several studies have reported that adult dermal stem cells exist in skin dermis and that these cells have properties similar to MSCs [11]–[22]. These dermal stem cells, which are considered important to maintaining skin homeostasis and for repairing damaged dermis, have been described in rodents and humans. Toma *et al* demonstrated that these cells, termed SKPs (skin-derived progenitors), are similar to embryonic neural crest stem cells and can differentiate into mesodermal lineage cells, such as adipocytes, osteoblasts and chondrocytes [11], [12]. Furthermore, these cells can acquire cell characteristics of non-mesodermal origin, including those of neural cells and hepatic cells. Another study found that multipotent fibroblasts in human dermis can be identified by using a single-cell clonal analysis [16]. Recently, we also reported that human dermal stem/progenitor cells (hDSPCs) from normal human dermal fibroblasts (NHDFs) can be enriched based on the ability to adhere to collagen type IV, which is a binding partner of CD29 [21], [22]. We demonstrated that these hDSPCs exhibit increased colony-forming efficiency compared with non -hDSPCs. In addition, we showed that the hDSPCs can differentiate into mesodermal and ectodermal cell types, implying that these cells are multipotent.

Sasaki *et al* previously showed that the transplantation of MSCs significantly improves wound healing in damaged mouse skin [23]. Other studies demonstrated that wound healing is enhanced when MSCs are administered to humans with acute skin wounds or with chronic skin wounds [24], [25]. However, in spite of the ability of MSCs to differentiate into specific cell lineages, the low levels of MSC engraftment after transplantation suggested that the beneficial effects of MSCs may be mediated more by their secretion of soluble factors, such as growth factors, than by their long-term presence in damaged tissue [26], [27]. A recent report has demonstrated that a conditioned medium culturing murine bone marrow-derived MSCs contains high levels of cytokines and is sufficient to stimulate macrophage and endothelial migration and improve wound healing in Balb/C mice [28].

We previously suggested the possible use of hDSPCs for acceleration of skin regeneration in aged or damaged skin. However, it is still not known whether hDSPCs can exert their beneficial effects on the regeneration of damaged tissues via paracrine mechanisms involving secretion of soluble factors such as growth factors. Therefore, in the present study, we first compared the levels of paracrine factors secreted from hDSPCs and non-hDSPCs and found that several growth factors, such as IGBP-1 and bFGF, were increased in hDSPC-derived conditioned medium (hDSPC-CM). We then investigated whether hDSPC-CM has an influence on UVA-irradiated NHDFs. We found that hDSPC-CM up-regulated the mRNA expression levels of collagen types I, IV and V and TIMP1, which were down-regulated by UVA irradiation and down-regulated the mRNA expression level of MMP1, which was up-regulated by UVA irradiation. We also showed that hDSPC-CM promoted *in vitro* wound healing of UVA-irradiated NHDFs. In addition, hDSPC-CM significantly decreased the number of UVA irradiation-induced apoptotic cells.

Our results suggest that hDSPC-CM provides another stem cell-based therapeutic potential for curing skin damaged by such harmful agents as UVA irradiation and oxidative stress.

## Materials and Methods

### Cell culture of NHDFs and enrichment of hDSPCs

Normal human dermal fibroblasts (NHDFs, Lonza, Basel, Switzerland) derived from the skin were cultured in DMEM (Lonza) containing 10% FBS, 100 U/ml penicillin and 100 μg/ml streptomycin at 37°C. The NHDFs were used within three passages.

Collagen type IV (Sigma-Aldrich, St. Louis, MO, USA)-coated dishes were prepared by coating 100-mm dishes with collagen type IV (20 μg/ml) overnight at 4°C. The NHDFs were plated onto the collagen type IV-coated dishes and then sorted on the basis of their ability to adhere to collagen type IV within 5 min (human Dermal Stem/Progenitor Cells; hDSPCs) or within 12 hr (non-hDSPCs) at 37°C [21], [22].

### Preparation of hDSPC-CM

hDSPCs (1×10^5^ cells/ml) were cultured in DMEM (Lonza) serum-free medium. The conditioned medium (CM) was collected after 48 hr of suspension culture in Hydrocell^TM^ dishes (Nunc, Paisley, UK), centrifuged at 300×*g* for 5 min and filtered through a 0.22-μm syringe filter (Millipore, Billerica, MA, USA).

### Ultraviolet A irradiation

Before UVA exposure, the NHDFs were washed with PBS and protected from drying by adding DMEM (Phenol red-free, Lonza) at 0.1 ml/cm^2^. UVA irradiation was performed using BIO-SUN (Vilber Lourmat, Torcy, France). Immediately after the UVA irradiation, the DMEM was aspirated and replaced with the conditioned medium. UVA irradiation doses were tested from 4 to 10 J/cm^2^, and 6 J/cm^2^ was used in the ensuing experiments.

### Cell proliferation assay

The NHDFs were plated at a density of 2×10^5^ cells/well in 6-well plates, and the proliferation of the NHDFs was measured using a CCK-8 assay (Dojindo, Rockville, MD, USA). After UVA irradiation, the cells were continuously cultured for 48 hr under each condition. CCK-8 solution (10 μl) was added to the cells in 1 ml DMEM and incubated for 1 hr at 37°C; the absorbance was then measured at 450 nm using a SpectraMax 190 microplate reader (Molecular Devices, Sunnyvale, CA, USA).

### RNA isolation and real-time RT-PCR

Total RNA was extracted with TRIzol (Invitrogen, Carlsbad, CA, USA), and the RNA concentration was determined using a NanoDrop spectrophotometer (Thermo Scientific, Fremont, CA, USA). To produce cDNA, 2 μg of RNA was reverse-transcribed using ReverTra Ace reverse transcriptase (Toyobo, Osaka, Japan); the reverse transcription was stopped by adding Tris-EDTA buffer (pH 8.0) to a total volume of 100 μl. Real-time RT-PCR was performed according to the manufacturer's instructions. Briefly, each 20 μl PCR mixture contained 10 μl 2× TaqMan® universal PCR Master Mix, 1 μl 20× of TaqMan® Gene Expression assay, and 50 ng cDNA. Real-time RT-PCR was performed using a 7500 Fast Real-Time PCR System (Applied Biosystems, Foster city, CA, USA). The TaqMan® Gene Expression Assay was purchased from Applied Biosystems. The cDNA samples were analyzed to determine the expression of the following: COL1A1, Hs00164004_m1; COL4A1, Hs00266237_m1; COL5A1, Hs00609088_m1; MMP1, Hs00899658_m1; and TIMP1, Hs00171558_m1. Human GAPDH (43333764F) (Applied Biosystems) was used for normalizing the variation in the cDNA quantities from different samples.

### Detection of apoptosis

To stain apoptotic cells, the cells were washed twice with PBS and then once with Annexin V binding buffer (BD Pharmingen, San Jose, CA, USA). The cells were stained for 15 min with Annexin V-FITC (BD Pharmingen). After washing with Annexin binding buffer, the slides were mounted in binding buffer. The cells were examined using an EVOS fl fluorescence microscope (Advanced Microscopy Group, Mill Creek, WA, USA).

An analysis of apoptosis was also performed using an Annexin V-FITC apoptosis detection kit I (BD Pharmingen) with flow cytometry. Briefly, the cells were harvested 24 hr after culturing with hDSPC-CM, washed with PBS, and stained with Annexin V-FITC and propidium iodide (PI) for 15 min at RT in the dark. Flow cytometry was performed using FACSAria II (Becton Dickinson, San Jose, CA, USA). The data analyses were performed using FACSDiva software.

### Scratch wound healing assay

NHDFs were seeded in 6-well plates at a density of 2×10^5^ cells/well in DMEM containing 10% FBS and cultured until 80∼90% confluence. The NHDFs were irradiated with UVA and then scraped with a 5-ml pipette tip to generate scratch wounds; the cells were washed twice with serum free-DMEM to remove cell debris. The cells were then incubated at 37°C for 48 hr with the conditioned medium. To record scratch wound closure, images were captured at 0, 24, and 48 hr time points in the same position using an Olympus IX71 microscope (Olympus, Southborough, MA, USA). To assess wound closure, the wound perimeter under each condition was traced. Each time point was normalized to the post-scratch day 0 image area and reported as the percent area closed.

### Human growth factor/cytokine antibody array

The Raybio® Human Cytokine/Growth Factor Antibody array I (RayBiotech, Noncross, GA, USA) was used to assay over 23 cytokines/41 growth factors in the supernatants of sorted cell cultures. The array membranes were incubated in blocking buffer for 30 min at room temperature (RT), and 1 ml of the conditioned medium was added per well, followed by incubation for 1 hr at RT. The membranes were washed five times in wash buffer at RT, and a biotin-conjugated antibody was added for 1–2 hr at RT. The membranes were washed again, and 2 ml of HRP-conjugated streptavidin was added for 2 hr, followed by the addition of detection buffer for 2 min. The membranes were then detected using the LAS 3000 chemiluminescence imaging system (Fujifilm Inc., Tokyo, Japan).

### Statistical analysis

The statistical analyses of the data were performed using a one-way analysis of variance (ANOVA). The results are expressed as the mean ± standard deviation of at least three independent experiments, and *p*<0.05 was considered significant.

## Results

### Profiles of growth factors/cytokines secreted from hDSPCs

**Figure 1:**
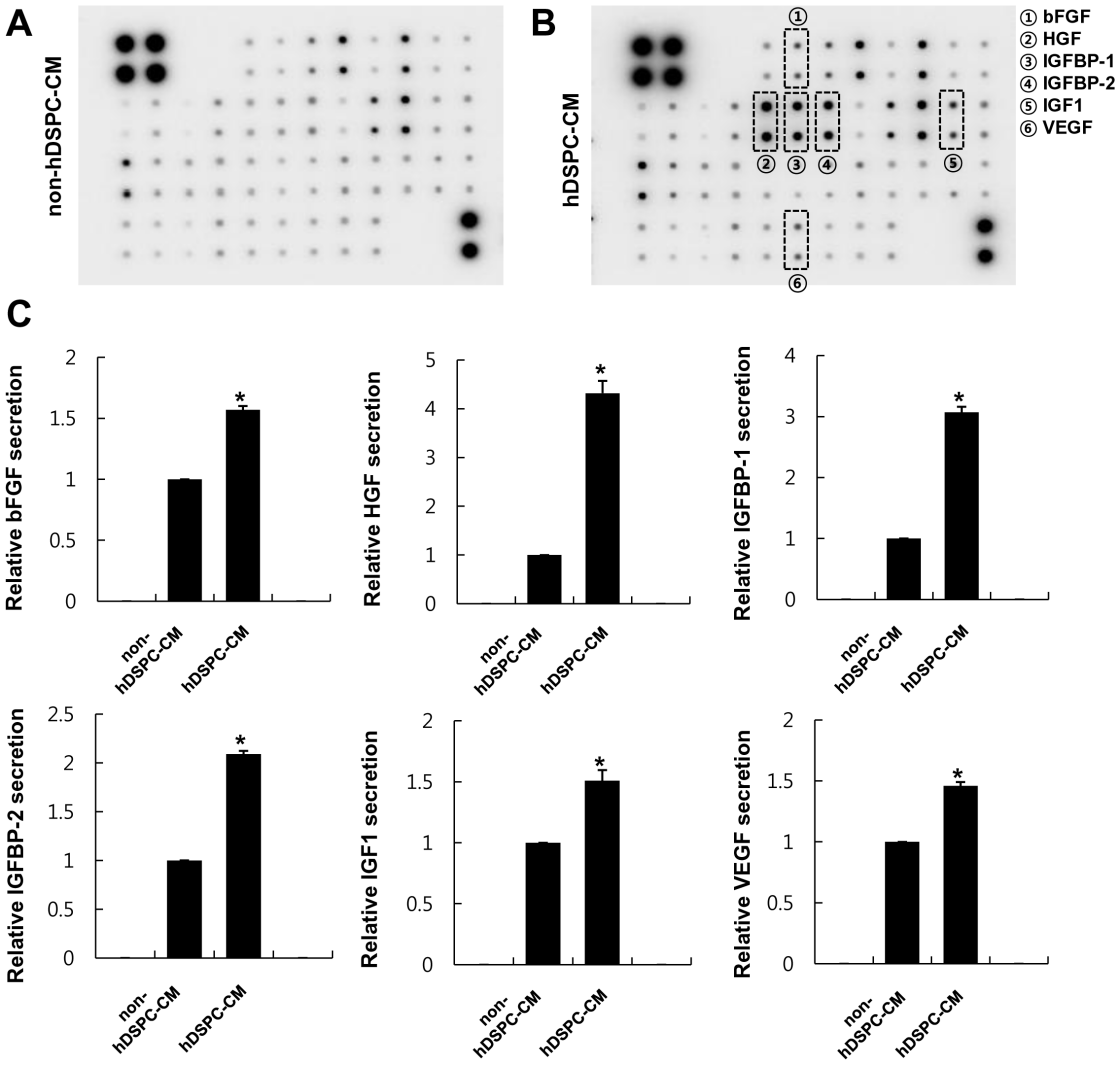
The secretion levels of specific growth factors were increased in hDSPC-CM. Growth factor secretion profiles of non-hDSPC-CM (A) and hDSPC-CM (B) using a human growth factor antibody array, respectively. Relative secretion levels of bFGF, HGF, IGFBP-1, IGFBP-2, IGF1, and VEGF in serum-free hDSPC-CM compared with non-hDSPC-CM (C). The graphs are shown as the mean ± S.D. of three independent experiments. *p<0.01.

According to previous reports [26]–[28], several types of mesenchymal stem cells, including adipose-derived stem cells, secrete a variety of growth factors and cytokines into their medium (conditioned medium). Thus, we examined the profiles of growth factors and cytokines secreted from hDSPCs using a human cytokine/growth factor antibody array. The results showed that hDSPCs secreted relatively higher levels of bFGF (1.56±0.03), HGF (4.32±0.25), IGFBP-1 (3.07±0.09), IGFBP-2 (2.09±0.03), IGF-1 (1.51±0.09), and VEGF (1.46±0.03) compared with non-hDSPCs (*p*<0.01) (Fig. 1, Table 1). However, we found that the hDSPCs showed no significant differences in their secretion level of such cytokines as IL-1α and IL-8 compared with the non-hDSPCs (Table S1).

**Table 1:** Relative growth factor secretion analysis of hDSPC-CM.

Name	Fold change (mean ± S.D)Ratio: hDSPC/non- hDSPC
AR (Amphiregulin)	1.11±0.03
bNGF (β-Nerve growth factor)	1.04±0.01
EGF (Epidermal growth factor)	1.17±0.01
EGF-R (Epidermal growth factor-receptor)	1.07±0.01
FGF4 (Fibroblast growth factor 4)	1.17±0.01
FGF6 (Fibroblast growth factor 6)	1.01±0.01
FGF7 (Fibroblast growth factor 7)	1.04±0.02
GCSF (Granulocyte colony stimulating factor)	1.35±0.13
GDNF (Glial cell-derived neurotrophic factor)	1.2±0.13
GM-CSF (Granulocyte-macrophage colony-stimulating factor)	1.1±0.05
HB-EGF (Heparin-binding EGF-like growth factor)	1.06±0.01
IGFBP-3 (Insulin-like growth factor-binding protein-3)	1.08±0.03
IGFBP-4 (Insulin-like growth factor-binding protein-4)	1.02±0.02
IGFBP-6 (Insulin-like growth factor-binding protein-6)	1.25±0.06
IGF-1 SR (Insulin-like growth factor-1 receptor)	1.07±0.02
IGF2 (Insulin-like growth factor2)	1.26±0.1
M-CSF (Macrophage colony stimulating factor)	1.18±0.03
M-CSF R (Macrophage colony stimulating factor receptor)	1.03±0.02
NT-3 (Neurotrohpin-3)	0.99±0.04
NT-4 (Neurotrohpin-4)	1.03±0.05
PDGF Rα (Platelet derived growth factor receptor α)	1.07±0.05
PDGF Rβ (Platelet derived growth factor receptor β)	1.07±0.07
PDGF-AA (Platelet derived growth factor-AA)	1.01±0.03
PDGF-AB (Platelet derived growth factor-AB)	0.91±0.06
PDGF-BB (Platelet derived growth factor-BB)	0.92±0.01
PIGF (Placental growth factor)	1.1±0.04
SCF (Stem cell factor)	0.93±0.01
SCF R (Stem cell factor receptor)	1.04±0.06
TGF-α (Transforming growth factor-α)	0.94±0.02
TGF-β (Transforming growth factor-β)	0.94±0.01
TGF-β2 (Transforming growth factor-β2)	0.98±0.05
TGF-β3 (Transforming growth factor-β3)	0.99±0.01
VEGF R2 (Vascular endothelial growth factor receptor2)	0.96±0.04
VEGF R3 (Vascular endothelial growth factor receptor3)	0.95±0.07
VEGF-D (Vascular endothelial growth factor-D)	0.95±0.03

The data shown are the mean ± S.D. of three independent experiments.

### Effects of hDSPC-CM on the mRNA expression levels of NHDF specific markers

**Figure 2:**
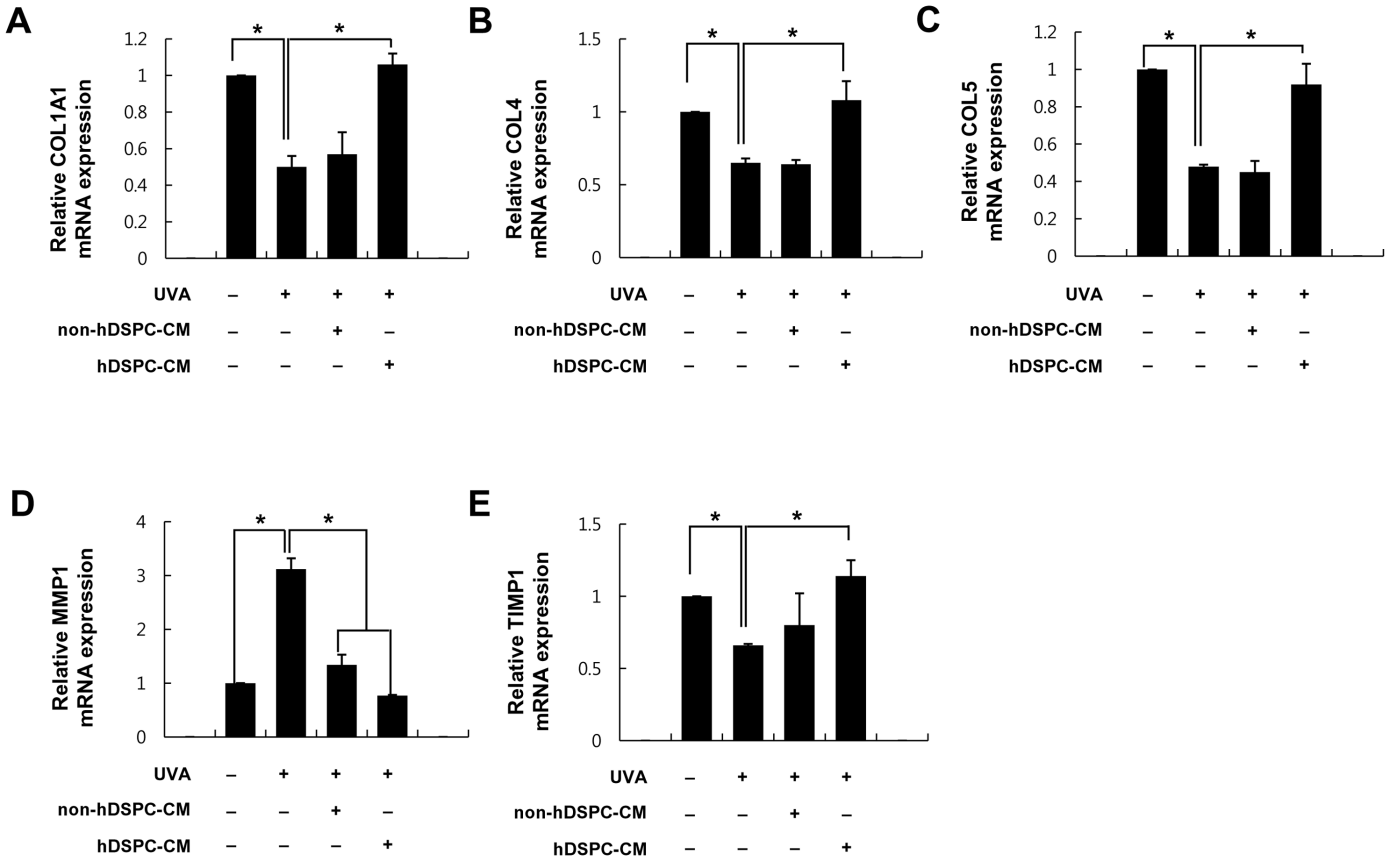
hDSPC-CM restored the down-regulated mRNA expressions of specific dermal makers in UVA-irradiated NHDFs. NHDFs were irradiated with UVA (6 J/cm^2^) and treated with either hDSPC-CM or non-hDSPC-CM for 24 hr. Total RNA was extracted, and real-time RT-PCR was performed for COL1A1(A), COL4A1(B), COL5A1(C), MMP1(D), and TIMP1(E). The graphs are shown as the mean ± S.D. of three independent experiments. *p<0.01

We next investigated whether hDSPC-CM or non-hDSPC-CM could restore the disturbed mRNA expression of NHDF specific markers in UVA-irradiated NHDFs. UVA irradiation (6 J/cm^2^) decreased the mRNA expression levels of collagen types I (0.5±0.06), IV (0.65±0.03), and V (0.48±0.01) and TIMP1 (0.66±0.01) (Fig. 2A–2C, 2E), which are among the most important components in skin dermis. Conversely, UVA irradiation increased the mRNA expression level of MMP 1 (3.12±0.2) (*p*<0.01) (Fig. 2D). Interestingly, both hDSPC-CM and non-hDSPC-CM significantly reduced the UVA-induced increase of MMP1 gene expression (Fig. 2D), whereas only hDSPC-CM significantly restored the down-regulated mRNA expression levels of collagen types I (1.06±0.06), IV (1.08±0.13), and V (0.92±0.11) and TIMP1 (1.14±0.11) by UVA irradiation (Fig. 2A–2C, 2E).

### Effects of hDSPC-CM on the wound healing process

**Figure 3:**
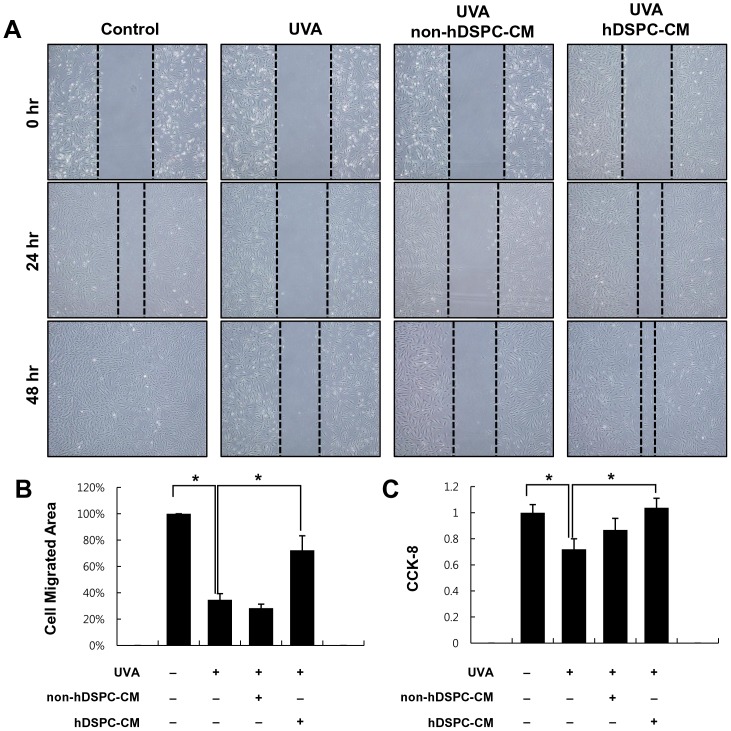
hDSPC-CM promoted the migration and proliferation of UVA-irradiated NHDFs. Effects of hDSPC-CM on NHDF migration. Scratch wound healing assays were performed using conditioned media and UVA-irradiated NHDFs for 48 hr. Images were obtained at 0, 24, and 48 hr (A). Quantitative analysis of the scratch wound healing assay after 48 hr (B). The proliferation of the NHDFs was examined in the presence or absence of hDSPC-CM or non-hDSPC-CM. CCK-8 assay was performed at 48 hr (C). The graphs are shown as the mean ± S.D. of three independent experiments. *p<0.01

To investigate whether hDSPC-CM has an effect on the migration of NHDFs irradiated with UVA (6 J/cm^2^), a scratch wound healing assay was performed. The data showed that the UVA-irradiated NHDFs exhibited significantly slower repair of scratch wounds compared with the control NHDFs (Fig. 3A, 3B). Although non-hDSPC-CM had no effect on the migration of UVA-irradiated NHDFs, hDSPC-CM significantly increased the migration of UVA-irradiated NHDFs, indicating that hDSPC-CM could improve the reduced migration of NHDFs irradiated with UVA (Fig. 3A, 3B). In addition, a CCK8 analysis also revealed that hDSPC-CM-treated NHDFs showed more recovery of reduced proliferation by UVA irradiation than non-hDSPC-CM-treated NHDFs, data that are consistent with the data from the scratch wound healing assay (Fig. 3C).

### Effects of hDSPC-CM on apoptotic NHDFs irradiated with UVA

**Figure 4:**
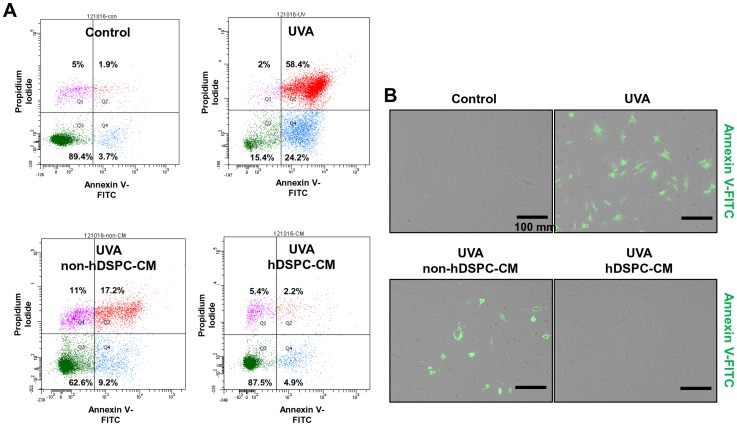
UVA irradiation-induced apoptotic cells were recovered by hDSPC-CM. NHDFs were irradiated with UVA (6 J/cm^2^) and incubated with either hDSPC-CM or non-hDSPC-CM for 24 hr and labeled with Annexin V-FITC and propidium iodide (PI). The distribution of apoptotic cells was analyzed using FACSAria II instrumentation. Only PI positive cells are dead (Q1). Cells showing Annexin V and PI double-labeling represent the stage of late apoptosis (Q2). Live cells were not labeled with Annexin V and PI (Q3), whereas Annexin V-labeled cells (Q4) represent the early stage of apoptosis. Ten thousand cells were analyzed for each condition (A). Apoptotic cells labeled with Annexin V under fluorescence microscopy to examine the effects of hDSPC-CM (B). The data are representative of three independent experiments.

FACS analyses were performed to estimate the effects of hDSPC-CM on the apoptotic cell death of the UVA-irradiated NHDFs. NHDFs were exposed to UVA at a dose of 6 J/cm^2^, incubated with hDSPC-CM or non-hDSPC-CM for 24 hr, and labeled with Annexin V-FITC and propidium iodide (PI). The FACS analysis revealed that, after UVA exposure, 24.2% of the UVA-irradiated cells (Annexin V-positive/PI-negative; Q4 region) were in the early apoptotic stage, which was significantly reduced to 4.9% in the hDSPC-CM-treated cells, similar to 3.7% for the control (Fig. 4A). The number of double-stained cells in the late stage of apoptosis (Annexin V-positive/PI-positive; Q2 region) was also significantly decreased in the hDSPC-CM-treated cells, from 58.4% to 2.2% compared with 17.2% for the non-hDSPC-CM-treated cells (Fig. 4A). The unlabeled cells, representing the live population (Annexin V-negative/PI-negative; Q3 region), were markedly increased in the hDSPC-CM-treated cells, from 15.4% to 87.5%, compared with 62.6% for the non-hDSPC-CM-treated cells (Fig. 4A). The fluorescent microscope images also showed that hDSPC-CM decreased the number of UVA-induced apoptotic cells, which were stained with Annexin V-FITC, compared with non-hDSPC-CM, data that were in accordance with the FACS analysis (Fig. 4B).

## Discussion

In the present study, we demonstrated that hDSPC-CM has several beneficial effects on NHDFs damaged by UVA irradiation. First, a real-time RT-PCR analysis revealed that hDSPC-CM restored the UVA-induced decrease of representative dermal markers, such as collagen types I, IV, and V and TIMP1, but also attenuated the UVA-induced increase of MMP1 in NHDFs (Fig. 2). Second, an *in vitro* scratch wound healing assay showed that hDSPC-CM enhanced the rate of wound closure in NHDFs irradiated with UVA compared with non-hDSPC-CM (Fig. 3). Third, the FACS analysis indicated that hDSPC-CM significantly decreased the number of NHDFs undergoing apoptotic cell death by UVA irradiation (Fig. 4).

Furthermore, when we applied the hDSPC-CM to NHDFs without UVA irradiation, we found that hDSPC-CM had no effects on expression levels of representative dermal markers (Fig. S1), migration (Fig. S2), the population of apoptotic cells (Fig. S3), and except for reduction of reactive oxygen species (ROS) level immediately after the treatment (Fig. S4), indicating that it is not easy to see the effects of hDSPC-CM on normal cells, although the hDSPC-CM has some helpful effects for the recovery of damaged cells.

The aging process causes a gradual decrease in the maintenance of both homeostasis and the regenerative properties of all tissues and organs [29]–[32]. In particular, upon skin aging through such processes as photoaging and intrinsic aging, the elasticity of skin is significantly reduced, the wrinkles in the human face gradually become visible and the capacity of wound healing gradually decrease [33]–[35]. These age-related changes may be due to a reduction in the function of adult stem cells, which exist in most tissues and are indispensible for normal tissue homeostasis, contributing to tissue repair and regeneration in response to damage [36]–[38].

Unlike UVB, UVA can penetrate into the lower dermis of skin and is largely involved in the photoaging mediated by oxidative stress [33]–[35]. Hydrogen peroxide is one of the reactive oxygen species (ROS) associated with UVA-induced cytotoxicity, as described previously [39], [40]. Several previous reports have suggested that the protective effects of stem cells on various types of cells against UVA-induced ROS generation may be due to the secretion of specific cytokines from the stem cells. For instance, it has been reported that HGF has a protective effect on retinal pigment epithelium in oxidative injury [41]. In addition, a few reports have demonstrated that bFGF reduces the epithelial cell death induced by hydrogen peroxide [42] and IGF-1 reduces oxidative damages by glucose and nicotine in fibroblasts [43]. In this study, although the underlying mechanisms regarding the protective effects of hDSPC-CM against UVA-induced cell damages were not elucidated, we presume that hDSPC-CM, which resulted in a higher expression of such growth factors as bFGF, IGF-1 and HGF (Fig. 1), may involve in cellular antioxidant pathways in the NHDFs and eventually inhibit the apoptotic cell death caused by UVA.

Wound healing is one of the most complex biological processes and requires a well-coordinated integration of cellular and molecular events of cell proliferation and migration, the redistribution of the extracellular matrix, angiogenesis, and tissue remodeling [44], [45]. Among the various types of cells involved in the wound healing process, fibroblasts are among the most important: in particular, fibroblasts in the dermis surrounding a wound proliferate rapidly and migrate to the wound area [44], [45]. After moving to the wound, fibroblasts begin to synthesize components of the extracellular matrix, such as collagen types I and III, which play a role in maintaining the integrity of the normal dermal environment. In this study, we found that hDSPCs specifically secreted higher levels of bFGF, IGFBP-1, IGFBP-2, HGF, VEGF and IGF-1 compared with non-hDSPCs (Fig. 1). Although we did not examine the effects of the identified growth factors on UVA-irradiated NHDFs, abundant supporting evidences suggest that these growth factors play important roles in the wound healing process. According to previous reports [46], [47], such growth factors as FGF and IGF-1 enhance the proliferation of fibroblasts and contribute to increasing the production of collagen I in fibroblasts. In addition, IGFBP plays an important role in skin homeostasis, in regulating the IGF-mediated signaling of dermal cell migration, and in proliferation [48]. Therefore, we suggest that both the recovery of the collagen types I, IV, and V and TIMP1 mRNA expression levels, which were down-regulated by UVA irradiation (Fig. 2), and the increase of the migratory properties of UVA-irradiated NHDFs treated with hDSPC-CM (Fig. 3) may be due to the specific growth factors secreted from the hDSPCs. Further experiments are required to confirm the secreted factor that are responsible for the protective and restoring effects of hDSPC-CM on UVA-damaged fibroblasts.

Late apoptotic cells are usually defined as Annexin V/PI-double positive, whereas early apoptotic cells are Annexin V-positive and PI-negative. Interestingly, the hDSPC-CM-treated cells showed a significantly decreased percentage of UVA-induced early and late apoptotic cells compared with the non-hDSPC-CM-treated cells (Fig. 4). Herein, we demonstrated that hDSPC-CM may possess the ability to enhance dermal fibroblast viability and proliferation after UVA irradiation. This enhanced viability and proliferation may be also due to the increased release of paracrine mediators, such as bFGF, IGFBP-1, IGFBP-2, HGF, VEGF and IGF-1, from hDSPCs compared with non-hDSPCs.

In conclusion, we suggest that hDSPC-CM ameliorated the UVA-induced damage of NHDFs in a paracrine fashion and that hDSPC-CM, containing specific secretory factors, may have a promise for treating photo-damaged skin.

## Supporting Information

Figure S1 **hDSPC-CM had no effects on mRNA expressions of specific dermal makers in NHDFs.** NHDFs were treated with either hDSPC-CM or non-hDSPC-CM for 24 hr. Total RNA was extracted, and real-time RT-PCR was performed for COL1A1(A), COL4A1(B), COL5A1(C), MMP1(D), and TIMP1(E). The graphs are shown as the means with error bars indicating S.D. of three independent experiments.
(TIF)

Figure S2 **hDSPC-CM had no effects on the migration and proliferation of NHDFs.** Effects of hDSPC-CM on NHDF migration. Scratch wound healing assays were performed using conditioned media for 48 hr. Images were obtained at 0, 24, and 48 hr (A). The proliferation of the NHDFs was examined in the presence or absence of hDSPC-CM or non-hDSPC-CM (B). The graphs are shown as the means with error bars indicating S.D. of three independent experiments.
(TIF)

Figure S3 **hDSPC-CM had no effects on cell death.** NHDFs were incubated with either hDSPC-CM or non-hDSPC-CM for 24 hr and labeled with Annexin V-FITC and propidium iodide (PI). The distribution of apoptotic cells was analyzed using FACSAria II instrumentation. Only PI positive cells are dead (Q1). Cells showing Annexin V and PI double-labeling represent the stage of late apoptosis (Q2). Live cells were not labeled with Annexin V and PI (Q3), whereas Annexin V-labeled cells (Q4) represent the early stage of apoptosis. Ten thousand cells were analyzed for each condition. Control cells (A), cells treated with non hDSPC-CM (B), and cells treated with hDSPC-CM (C) are shown. The data are representative of three independent experiments.
(TIF)

Figure S4 **hDSPC-CM reduced the level of H_2_O_2_ immediately after the treatment.** Fluorescence signals from Amplex Red assays, which are used to detect H_2_O_2_, in the presence or absence of UVA irradiation using assay buffer or conditioned media from either non-hDSPCs or hDSPCs (A, B) Absorption spectra after irradiation for 0 min (A, B) and 10 min (C, D). The graphs are shown as the mean ± S.D. of three independent experiments. *p<0.01
(TIF)

Table S1 **Relative cytokine secretion analysis of hDSPC-CM.**
(DOCX)
