# Supplementary figures and images for: Human Dermal Stem/Progenitor Cell-Derived Conditioned Medium Ameliorates Ultraviolet A-Induced Damage of Normal Human Dermal Fibroblasts
Source: PLoS One. 2013 Jul 11;8(7):e67604. doi: 10.1371/journal.pone.0067604 (PMC3708938; doi:10.1371/journal.pone.0067604)

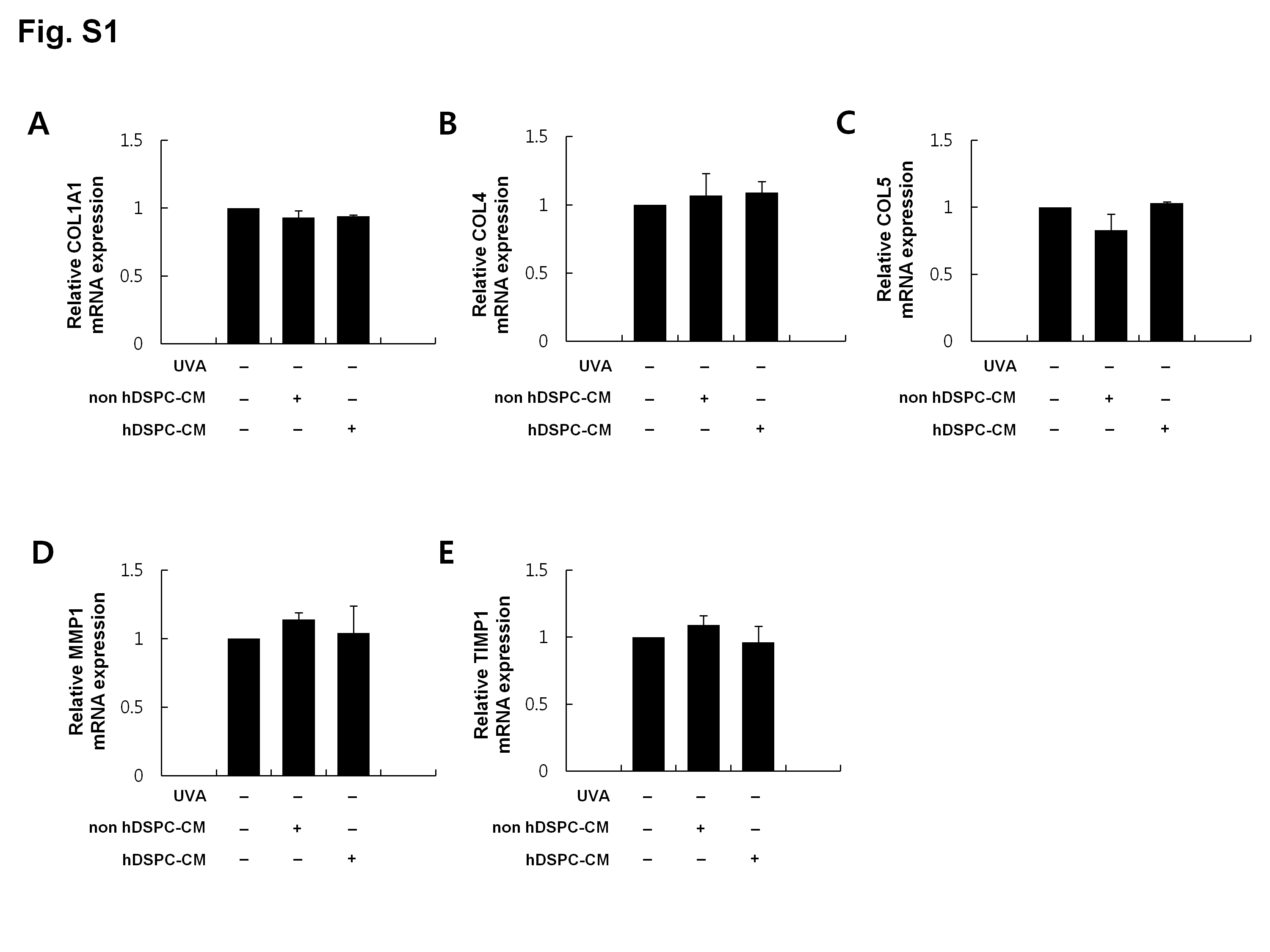

Supplement: Figure S1 — hDSPC-CM had no effects on mRNA expressions of specific dermal makers in NHDFs. NHDFs were treated with either hDSPC-CM or non-hDSPC-CM for 24 hr. Total RNA was extracted, and real-time RT-PCR was performed for COL1A1(A), COL4A1(B), COL5A1(C), MMP1(D), and TIMP1(E). The graphs are shown as the means with error bars indicating S.D. of three independent experiments. (TIF) [file pone.0067604.s001.tif]

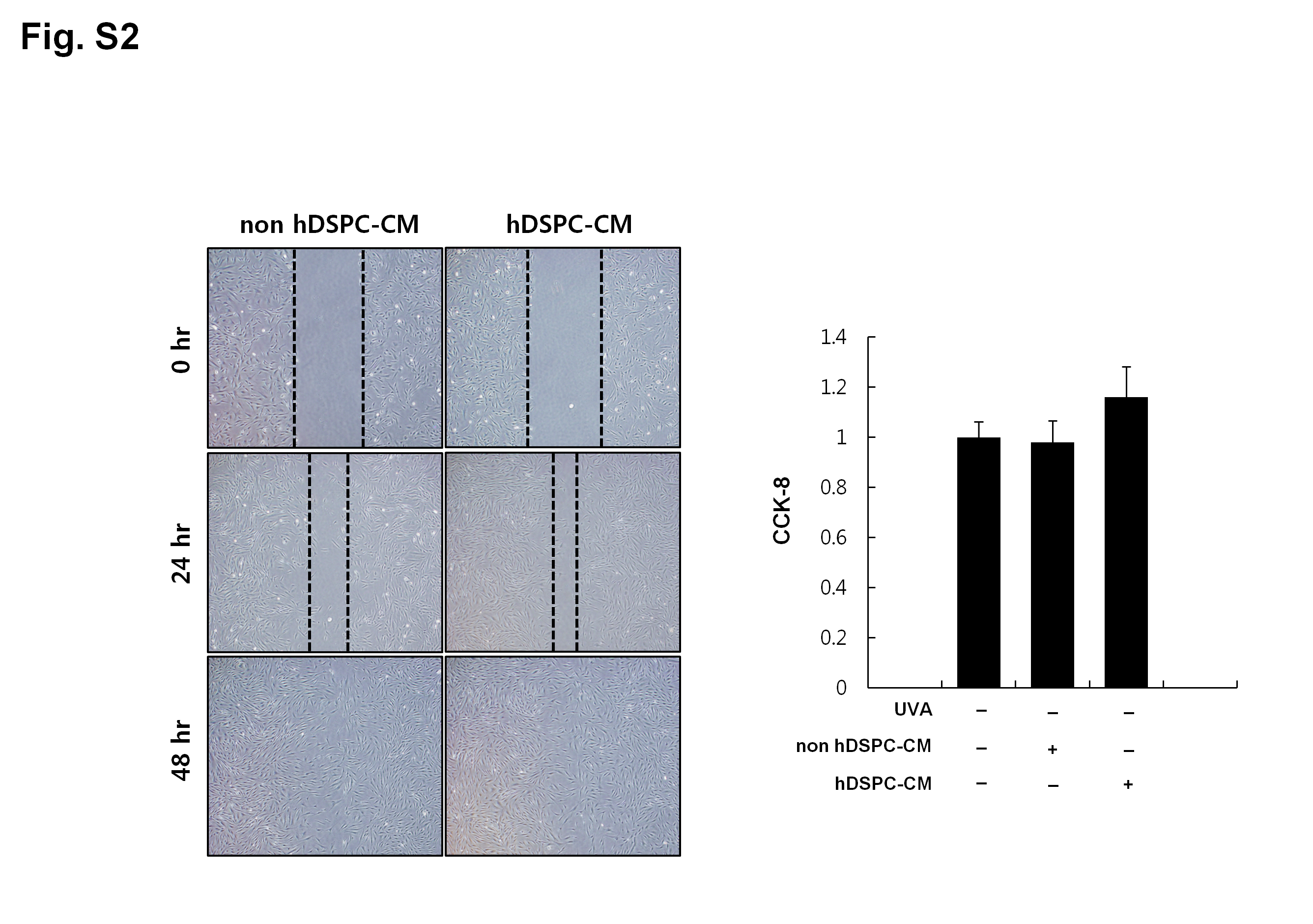

Supplement: Figure S2 — hDSPC-CM had no effects on the migration and proliferation of NHDFs. Effects of hDSPC-CM on NHDF migration. Scratch wound healing assays were performed using conditioned media for 48 hr. Images were obtained at 0, 24, and 48 hr (A). The proliferation of the NHDFs was examined in the presence or absence of hDSPC-CM or non-hDSPC-CM (B). The graphs are shown as the means with error bars indicating S.D. of three independent experiments. (TIF) [file pone.0067604.s002.tif]

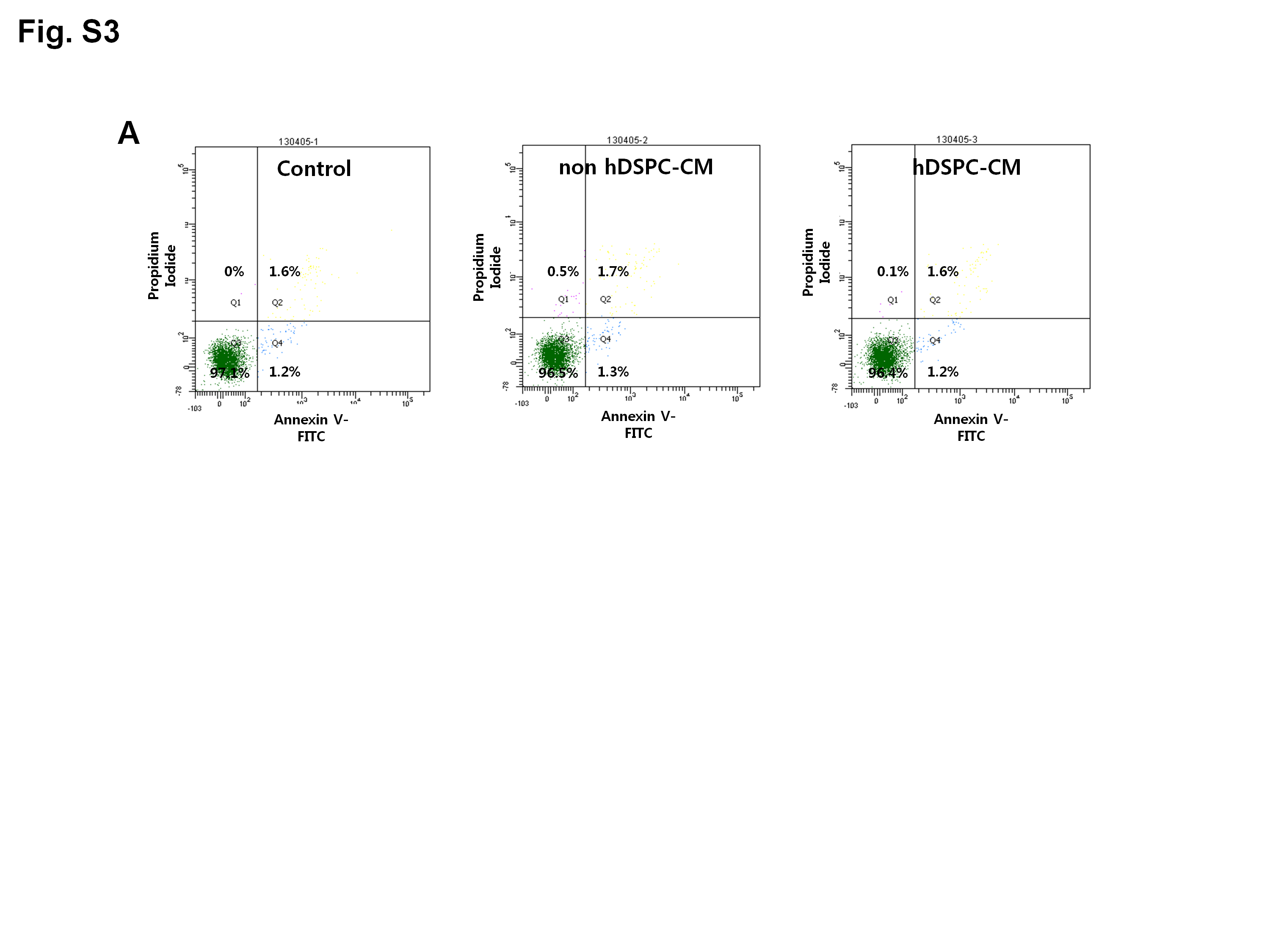

Supplement: Figure S3 — hDSPC-CM had no effects on cell death. NHDFs were incubated with either hDSPC-CM or non-hDSPC-CM for 24 hr and labeled with Annexin V-FITC and propidium iodide (PI). The distribution of apoptotic cells was analyzed using FACSAria II instrumentation. Only PI positive cells are dead (Q1). Cells showing Annexin V and PI double-labeling represent the stage of late apoptosis (Q2). Live cells were not labeled with Annexin V and PI (Q3), whereas Annexin V-labeled cells (Q4) represent the early stage of apoptosis. Ten thousand cells were analyzed for each condition. Control cells (A), cells treated with non hDSPC-CM (B), and cells treated with hDSPC-CM (C) are shown. The data are representative of three independent experiments. (TIF) [file pone.0067604.s003.tif]

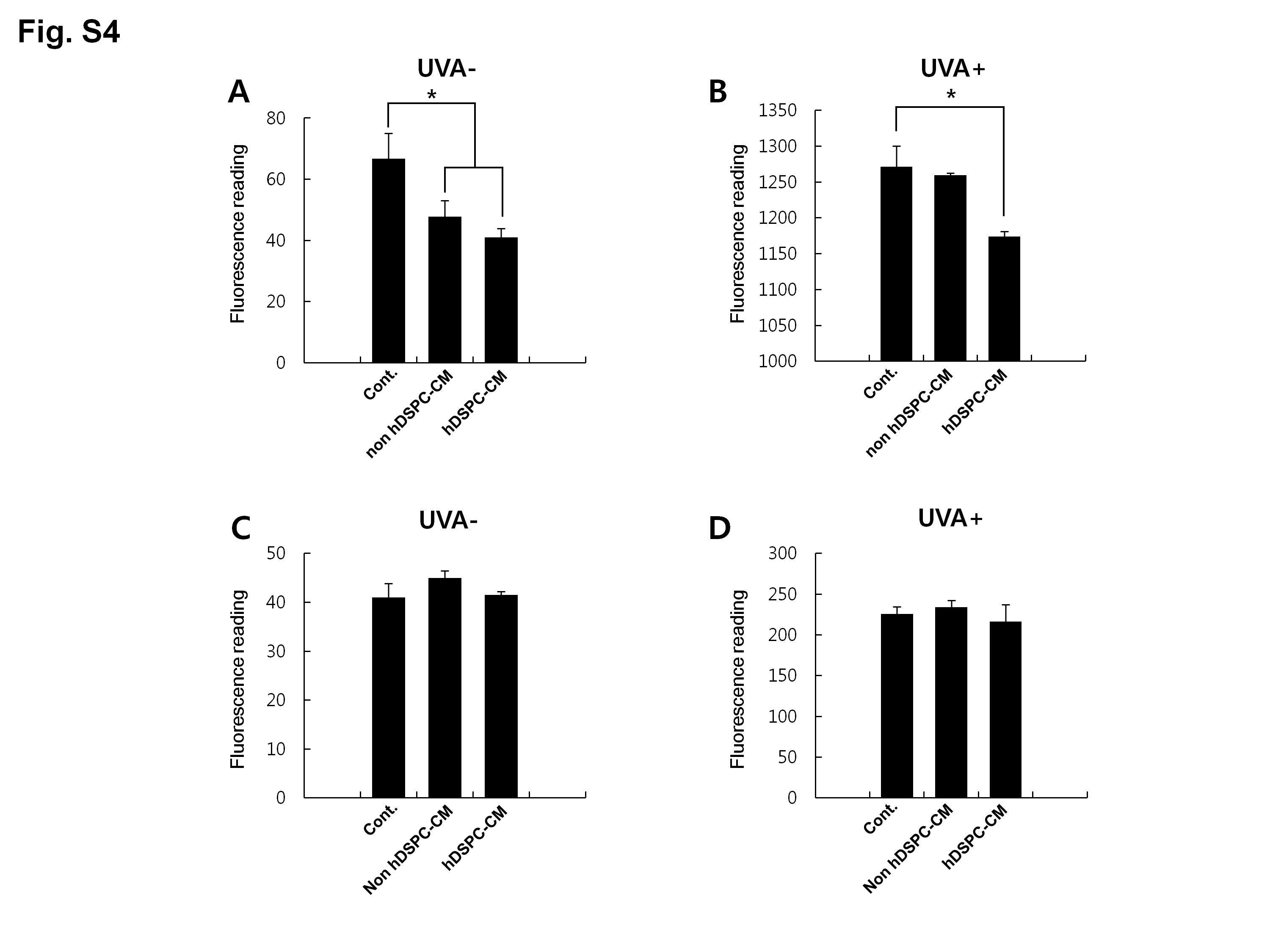

Supplement: Figure S4 — hDSPC-CM reduced the level of H2O2 immediately after the treatment. Fluorescence signals from Amplex Red assays, which are used to detect H2O2, in the presence or absence of UVA irradiation using assay buffer or conditioned media from either non-hDSPCs or hDSPCs (A, B) Absorption spectra after irradiation for 0 min (A, B) and 10 min (C, D). The graphs are shown as the mean ± S.D. of three independent experiments. *p<0.01 (TIF) [file pone.0067604.s004.tif]
