# Supplementary material for: Human Dermal Stem/Progenitor Cell-Derived Conditioned Medium Ameliorates Ultraviolet A-Induced Damage of Normal Human Dermal Fibroblasts
Source: PLoS One. 2013 Jul 11;8(7):e67604. doi: 10.1371/journal.pone.0067604 (PMC3708938; doi:10.1371/journal.pone.0067604)
Supplement: Table S1 — Relative cytokine secretion analysis of hDSPC-CM. (DOCX) [file pone.0067604.s005.docx]

**Table S1. Relative cytokine secretion analysis of hDSPC-CM**

The data shown are the mean ± S.D. of three independent experiments.

| **Name** | **Fold change (mean ± S.D)**  **Ratio: hDSPCs/non hDSPCs** |
| --- | --- |
| GCSF (Granulocyte colony-stimulating factor) | 0.99 ± 0.02 |
| GM-CSF (Granulocyte-macrophage colony-stimulating factor ) | 1.01 ± 0.01 |
| GRO (Growth-related oncogene) | 1.15 ± 0.12 |
| GRO-α (Growth-related oncogene-α) | 1.02 ± 0.01 |
| IL-1α (Interleukin-1α) | 0.98 ± 0.02 |
| IL-2 (Interleukin-2) | 0.98 ± 0.01 |
| IL-3 (Interleukin-3) | 1.03 ± 0.02 |
| IL-5 (Interleukin-5) | 1.03 ± 0.01 |
| IL-6 (Interleukin-6) | 1.02 ± 0.1 |
| IL-7 (Interleukin-7) | 1.02 ± 0.01 |
| IL-8 (Interleukin-8) | 0.90 ± 0.01 |
| IL-10 (Interleukin-10) | 1.03 ± 0.01 |
| IL-13 (Interleukin-13) | 1.01 ± 0.02 |
| IL-15 (Interleukin-15) | 1.00 ± 0.02 |
| INF-γ (Interferon-γ) | 1.08 ± 0.01 |
| MCP-1 (monocyte chemoattractant protein-1) | 1.11 ± 0.1 |
| MCP-2 (monocyte chemoattractant protein-2) | 1.01 ± 0.02 |
| MCP-3 (monocyte chemoattractant protein-3) | 1.00 ± 0.01 |
| MIG (Monokine induced by –interferon) | 1.02 ± 0.04 |
| RANTES (Regulated upon activation, normal T-cell expressed and presumably secreted) | 1.08 ± 0.05 |
| TNF-α (Tumor necrosis factor-α) | 1.04 ± 0.01 |
| TNF-β (Tumor necrosis factor-β) | 1.06 ± 0.02 |
